# Supplementary material for: Antitumor Effect of Cabozantinib in Bone Metastatic Models of Renal Cell Carcinoma
Source: Biology (Basel). 2021 Aug 16;10(8):781. doi: 10.3390/biology10080781 (PMC8389553; doi:10.3390/biology10080781)
Supplement: Supplementary file 1 [file biology-10-00781-s001.zip › biology-1309689-supplementary.pdf]

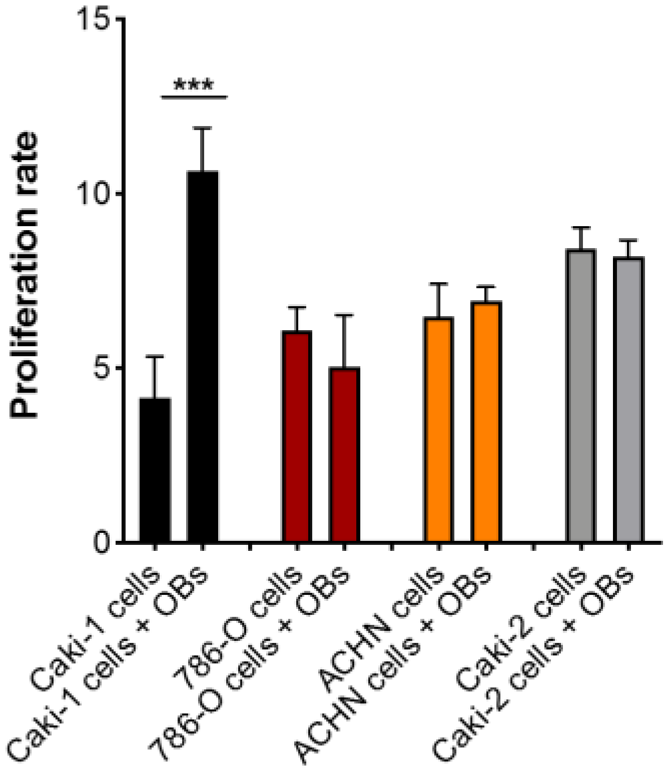

Supplementary Figure 1. Effect of OBs on metastatic RCC cell proliferation. Proliferation rate analysis of Caki-1 GFP+, 786-O GFP+, ACHN GFP+ and Caki-2 GFP+ cells in monoculture or co-cultured with OBs. Data are expressed as mean ± SD; *** $p<0.001$.

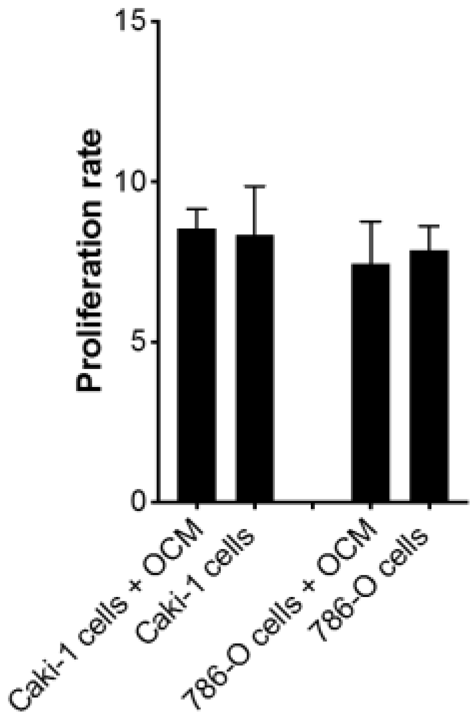

Supplementary Figure 2. Proliferation rate analysis of Caki-1 GFP+ and 786-O GFP+ cells in monoculture or in presence of OB conditioned media (OCM).
